# Bacterial Resistance Toward Antimicrobial Ionic Liquids Mediated by Multidrug Efflux Pumps

**DOI:** 10.3389/fmicb.2022.883931

**Published:** 2022-05-19

**Authors:** Tobias Gundolf, Roland Kalb, Peter Rossmanith, Patrick Mester

**Affiliations:** ^1^Christian Doppler Laboratory for Monitoring of Microbial Contaminants, Unit for Food Microbiology, Department of Veterinary Public Health and Food Science, University of Veterinary Medicine, Vienna, Austria; ^2^Proionic Production of Ionic Substances GmbH, Grambach, Austria; ^3^Joint BioEnergy Institute, Lawrence Berkeley National Laboratory, Berkeley, CA, United States; ^4^Unit for Food Microbiology, Department of Veterinary Public Health and Food Science, University of Veterinary Medicine, Vienna, Austria

**Keywords:** ionic liquids, antimicrobial, multidrug resistance, efflux pump, Resistance Nodulation Division, structure–activity relationship, *Escherichia coli*, *Salmonella enterica*

## Abstract

The effective elimination of foodborne pathogens through cleaning and disinfection measures is of great importance to the food processing industry. As food producers rely heavily on disinfectants to control pathogenic bacteria in their facilities, the increasing spread of tolerant, often even multidrug resistant, strains is of particular concern. In addition to efforts to prevent or at least reduce development and spread of strains resistant to disinfectants and sanitizers, there is an urgent need for new and effective antimicrobials. One new class of promising antimicrobials is ionic liquids (ILs), which have been reported to be effective against resistant strains as they interact with bacterial cells in multiple ways, but investigations of their effectivity against MDR bacteria or specific defense mechanisms are still limited. This study investigates the role of multidrug efflux pumps of the Resistance Nodulation-Division family (RND) on the resistance of bacterial pathogens *Escherichia coli* and *Salmonella enterica* serovar Typhimurium toward 10 antimicrobial active ILs. Results reveal that, while known structure–activity relationships (SARs), such as the side-chain effect, were found for all strains, antimicrobial ILs with one elongated alkyl side chain were significantly affected by the RND efflux pump, highlighting the importance of efflux pumps for future IL toxicity studies. In case of antimicrobial ILs with multiple side chains and different cationic head groups, two ILs were identified that were highly active against all investigated strains with little to no effect of the efflux pump. The results obtained in this study for RND efflux pumps can serve as a starting point for identifying and designing antimicrobial ILs as effective biocides against MDR bacteria.

## Introduction

Humanity’s history is a continuous battle between us and microbial pathogens and for the most part, we were on the losing side with bacterial and viral infections being among the major causes of morbidity and mortality worldwide. Thanks to the development and improvement of sanitation, hygiene practices and especially discovery of disinfectants, antibiotics, and vaccinations since the early 20th century, deaths from infectious diseases have declined markedly. This decline can be considered one of the biggest success stories in human history (CDC on infectious diseases in the United States: 1900-99, 1999) but is under pressure due to a dramatic increase of multidrug-resistant species (Andersson and Hughes, 2014; Qiao et al., 2018). Disinfectants play an important role in maintaining acceptable health standards by significantly reducing microbial loads as well as reducing, if not eliminating, pathogens (Tezel and Pavlostathis, 2015). Quaternary ammonium compounds (QAC) are among the most commonly used disinfectants in a variety of different industries including hospital, water, cosmetic, and the food industry, where bacteria are often exposed to disinfectants through the entire food chain (Buffet-Bataillon et al., 2016; Martínez-Suárez et al., 2016; Duze et al., 2021). Upon continued exposure, bacteria can adapt to biocides, a phenomenon known as biocide resistance (Poole, 2002; Andersson and Hughes, 2014; Grande Burgos et al., 2016; Gadea et al., 2017), which can increase the ability of pathogens to persist in food environments (Martínez-Suárez et al., 2016; Chmielowska et al., 2021; Guidi et al., 2021) and can be transferred from one species to another in the food environment (Hansen et al., 2007; Szmolka and Nagy, 2013). Bacteria can elicit non-specific mechanisms of resistance mediated by efflux pumps, which can accommodate a diversity of chemical structures as substrates including biocides and antibiotics (Poole, 2002, 2004, 2005, 2007; Grande Burgos et al., 2016; Shafaati et al., 2016).

Bacterial efflux pumps actively transport many antimicrobials and/or antibiotics out of the cell and are major contributors to the intrinsic resistance of bacteria (Putman et al., 2000; Tezel and Pavlostathis, 2015; Dam et al., 2018). While some efflux pumps have narrow substrate specificity, many transport a wide range of structurally dissimilar substrates and are known as multidrug resistance (MDR) efflux pumps. There are well-studied examples of MDR efflux pumps that are present in all bacteria, and new pumps that export antibiotics continue to be described. There are five classes of MDR efflux pumps: the ATP-binding cassette family (ABC), the Major Facilitator Superfamily (MFS), the Multidrug and Toxic Compound Extrusion family (MATE), the Resistance Nodulation-Division family (RND), and the Small-Multidrug Resistance family (SMR). Family division is based on the number of structural components that compromise each pump, the number of membranes they span, their substrate specificity, and the energy source used (Putman et al., 2000; Tezel and Pavlostathis, 2015). Whereas ABC, MATE, MFS, and SMR efflux pumps are widely distributed in both Gram-negative and Gram-positive species, the RND transporter type is exclusively found in Gram-negative bacteria, as it forms a tripartite complex that span from the inner to the outer membranes and is one of the best-characterized clinically relevant MDR efflux transporter type. Well-studied examples include the multidrug efflux pump AcrB in *Escherichia coli* and MexB in *Pseudomonas aeruginosa*. RND pumps, such as AcrB, are homo-trimers that reside in the inner membrane and form a tripartite complex with a periplasmic adaptor protein, such as AcrA and an outer-membrane channel, such as TolC (Chetri et al., 2019). Collectively, it is clear that there is an urgent need to identify novel antibacterial agents and biocides to combat the plethora of resistant bacterial genotypes (Andersson and Hughes, 2014; Bodro et al., 2014; Qiao et al., 2018; Prudêncio et al., 2020).

One promising new chemical class in this regard has been ionic liquids (ILs). Ionic liquids, defined as organic salts with a melting points below 100°C (Wasserscheid and Welton, 2008), have attracted substantial attention from both academia and industry due to their unique physiochemical properties and high tuneability (Rogers and Seddon, 2003; Aschenbrenner et al., 2009; Cevasco and Chiappe, 2014) including applications in medicine and as pharmaceuticals. In this context, ILs are applied in mostly one of two ways. Either as an antimicrobial active agent itself, as components of drug or drug delivery systems and as solvents in drug synthesis (Ferraz et al., 2011; Prudêncio et al., 2020). So-called API-ILs facilitate the incorporation of active pharmaceutical ingredients (API) into an IL form (Hough et al., 2007; Ferraz et al., 2011; Mester et al., 2016; Bromberger et al., 2020). One advantage of ILs, in comparison to other antimicrobials, is that they act on bacterial cells in multiple ways. Previous studies have demonstrated that ILs are (i) interacting with bacterial membrane and wall (Mester et al., 2016; Borkowski et al., 2017); (ii) disrupting cell integrity (Venkata Nancharaiah et al., 2012; Cook et al., 2019); (iii) destabilizing proteins and hindering their enzymatic activity (Mester et al., 2019; Tarannum et al., 2019; Bromberger et al., 2020); (iv) dysregulating bacterial metabolism (Yu et al., 2016; Cłapa et al., 2021); (v) triggering oxidative stress response (Yu et al., 2016); and (vi) leading to DNA damage (Kowalczyk et al., 2018). Consequently, ILs can be considered versatile antimicrobials of great potential with a wide spectrum of antibacterial mechanisms, thus potentially having an advantage against MDR resistant bacteria.

Nevertheless, there is still only a very limited amount of studies investigating the effectivity against MDR bacteria and to understand the impact of different bacterial defense strategies and mechanisms. What could be demonstrated in previous studies is a significant difference of antimicrobial IL efficacy between Gram-positive and Gram-negative bacteria. This difference is mainly attributed to the presence of outer membrane and lipopolysaccharide (LPS) layer in Gram-negative microorganisms (Cole et al., 2011; Weyhing-Zerrer et al., 2017). Due to its hydrophilic nature, LPS can prevent large, hydrophobic compounds from passing through the membrane and the impact of the LPS structure on overall IL susceptibility has been previously demonstrated (Gundolf et al., 2018; Kowalczyk et al., 2018). Interestingly, the influence of bacterial efflux pumps, one of the most important resistance mechanism toward antimicrobials as well as antibiotics, on antimicrobial IL efficacy has been scarcely investigated up to this point. Concerning antimicrobial ILs, only few studies investigated the impact of efflux pumps on bacterial resistance. For the Gram-positive pathogen *Listeria monocytogenes*, the SMR transporter QacH was shown to significantly increase the resistance of bacterial cells against classic QAC-based biocides as well as ILs with long alkyl side chains (Mester et al., 2015). For the Gram-negative bacterium *Enterobacter lignolyticus*, an efficient transport of [C_2_mim]^+^ cations outside the cell was found for efflux pump from a MFS encoded by the *eilA* gene (Ruegg et al., 2014).

Taken together, it is clear that the role of bacterial efflux pumps in regard to antimicrobial active ILs is understudied. Consequently, the aim of our study was to determine the impact of some of the most important efflux pump types on susceptibility of bacterial pathogens to antimicrobial ILs, which could subsequently influence the future design of these substances. To accomplish this objective, we investigated the impact of the multidrug efflux pump belonging the RND on the resistance of the bacterial pathogens *E. coli* and *Salmonella enterica* serovar Typhimurium toward a set of 10 antimicrobial active ILs.

## Materials and Methods

### Ionic Liquids and Other Chemical Substances

QACs: benzalkonium chloride (BC), benzethonium chloride (BZ), cetylpyridinium chloride (CP), cetyltrimethylpyridinium chloride (CTAB), and domiphen bromide (DB) were purchased from Sigma-Aldrich (Steinheim, Germany).

The ILs used in this study were either (**a**) provided by Proionic GmbH (Grambach, Austria) with a nominal purity of >98%, (**b**) purchased from Iolitec (Ionic Liquid Technologies GmbH, Heilbronn, Germany) with a nominal purity of >98%, or (**c**) synthesized in our laboratory, according to the CBILS© route (CBILS is a registered trademark of Proionic GmbH; Kalb et al., 2005, 2016). The following ILs were investigated as: ILs with one elongated alkyl side chain 1-decyl-3-methylimidazolium chloride ([C_10_mim][Cl];**a**), 1-dodecyl-3-methylimidazolium chloride ([C_12_mim][Cl];**b**), 1-methyl-3-tetradecylimidazolium chloride ([C_14_mim][Cl];**b**), 1-hexadecyl-3-methylimidazolium chloride ([C_16_mim][Cl];**b**) trimethyldecylammonium chloride ([TMC_10_A][Cl];**c**), trimethylhexadecylammonium chloride ([TMC_16_A][Cl];**c**). ILs with two elongated alkyl side chains dioctyldimethylammonium chloride ([DC_8_DMA][Cl];**c**) and 1-3-didecyl-2-imidazolium chloride ([C_10_C_10_im][Cl];**b**). ILs with three elongated alkyl side chains trioctylmethylammonium chloride ([TC_8_MA][Cl];**a**) and trioctylmethylphosphonium chloride ([TMC_10_P][Cl];**c**).

### Bacterial Strains and Culture Conditions

*Escherichia coli* BW25113 (wild-type), *E. coli* JW0451-2 (Δ*acrB*), *E. coli* JW0452-3 (Δ*acrA*), and *E. coli* JW5503-1 (Δ*tolC*) were obtained from the Coli Genetic Stock Centre (CGSC, Yale University) and are part of the Keio collection of *E. coli K-12* single-gene knockout mutants (Baba et al., 2006). The Keio collection contains a set of precisely defined, single-gene deletions of all nonessential genes in *E. coli* K-12, which enables systematic analyses of unknown gene functions and gene regulatory networks but also for genome-wide testing of mutational effects in a common strain background, *E. coli* K-12 BW25113. Open-reading frame coding regions were replaced with a kanamycin cassette flanked by FLP recognition target sites by using a one-step method for inactivation of chromosomal genes and primers designed to create in-frame deletions upon excision of the resistance cassette (Baba et al., 2006). *S. enterica* serovar Typhimurium ATCC 14028s (wild-type) and *S. enterica* serovar Typhimurium NKS148 (Δ*acrB*) were kindly provided by Kunihiko Nishino (Osaka University; Horiyama et al., 2010; Yamasaki et al., 2013). A detailed description of the respective strains is provided in the supplement section (Supplementary Table S1). Bacterial strains were grown overnight in tryptone soy broth supplemented with 0.6% (w/v) yeast extract (Oxoid™, Hamsphire, United Kingdom) and 30 μg/ml kanamycin at 37°C, with the exception of the wild-type strain *E. coli* BW25113 and the *S. enterica* strains that lack the kanamycin resistance cassette. Twenty-four hours growth curves were performed in 96-well microtiter plates (Corning B.V Life Sciences, Amsterdam, Netherlands) on measuring their optical densities every hour at a wavelength of 610 nm in a TECAN F100 microplate reader (Tecan Austria GmbH., Groeding, Austria). Bacteria were maintained at −80°C using Microbank™ technology (Pro-Lab Diagnostics, Richmond Hill, Canada).

### Minimal Inhibitory Concentration Assessment

MICs of the test chemicals (ILs, QACs, NaCl, KH_2_PO_4_, ethanol, and urea as well as pH) were assessed by applying the serial 2-fold dilution microtiter plate method in TSB-Y medium (Morrissey et al., 2009). In order to create a constant cell status for each experiment, 1 ml aliquots of the respective overnight cultures were transferred into 9 ml of fresh TSB-Y medium (1:10 dilution) and incubated for 3 h at 37°C to ensure that cells were in a logarithmic growth phase. Subsequently, each well, which contained a serial diluted antimicrobial substance (dilution 1:2), was inoculated with 5 × 10^5^ CFU of the respective bacterial cells. After inoculation with the respective bacteria, absorbance of the 96-well microtiter plates (Corning B.V Life Sciences, Amsterdam, Netherlands) was measured at a wavelength of 610 nm in a TECAN F100 microplate reader (Tecan Austria GmbH, Groeding, Austria) to monitor for any possible interference by the antimicrobial substances. The microtiter plates were then incubated for 24 h at the 37°C and bacterial growth assessed by measuring the absorbances at 610 nm. The MIC was defined as the lowest concentration of the tested antimicrobial substance where no bacterial growth could be measured after 24 h. Results are presented as mean MICs and upper and lower limits of 95% CIs of at least three experiments performed on different days. Each experiment included positive (bacterial growth control without ILs) and negative controls (medium without the addition of bacteria).

## Results and Discussion

### Influence of Efflux Pumps on Bacterial Growth and Susceptibility to Chemical Substances

**Figure 1:**
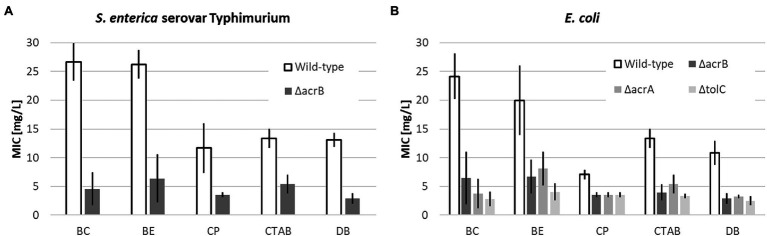
Mean minimal inhibitory concentration (MIC) values for quaternary ammonium compounds (QACs; BC, BE, CP, CTAB, and DB) including upper and lower limits of 95% CIs of the respective *Salmonella enterica* **(A)** and *Escherichia coli* **(B)** strains after 24 h incubation at 37°C.

This study investigates the role of efflux pumps deletions on the susceptibility of four *E. coli* strains (wild type and three different single-gene deletion mutants), as well as Salmonella NKS (Figure 1A) toward antimicrobial ILs. For *E. coli*, three efflux pump deletion strains (Δ*acrA*, Δ*acrB*, and Δ*tolC*) are missing one part of the tripartite complex of the RND transporter family. The three different mutant strains were included in this study to investigate possible differences concerning IL susceptibility if only one part of the tripartite complex is missing. For *S. enterica*, two strains (wild type and RND deletion mutant Δ*acrB*) were studied in order to investigate possible susceptibility differences between the two species.

To investigate if efflux pump deletion affects bacterial growth, the growth of wild type and deletions mutants was monitored for 24 h while measuring cell density hourly. No significantly impaired growth was observed for any of the four deletion strains compared to their respective wild type (data not shown). Further, strains were subjected to six hydrophilic chemical substances as an additional control to test strain viability/vitality. No significant differences between the wild-type strains and the deletion mutants were found indicating that the efflux strains were *per se* not less robust compared to the wild-type strains and the results for QACS and ILs can be interpreted accordingly (Table 1).

**Table 1:** Mean MIC values (bold) for chemicals including upper and lower limits of 95% CIs (in brackets) of the respective Salmonella enterica and Escherichia coli strains after 24 h incubation at 37°C.

	*S. enterica*		*E. coli*			
	Wild-type	**∆** *acrB*	Wild-type	**∆** *acrB*	**∆** *acrA*	**∆** *tolC*
Methanol (%; v/v)	**4.7**	**3.9**	**9.4**	**7.8**	**6.3**	**4.7**
	(4.7)	(2.4–5.4)	(9.38)	(4.8–10.9)	(3.2–9.3)	(4.7)
Ethanol (%; v/v)	**3.8**	**4.5**	**4.5**	**4.5**	**4.5**	**3.8**
	(2.3–5.2)	(4.5)	(4.5)	(4.5)	(4.5)	(2.3–5.2)
KH_2_PO_4_ (%; w/v)	**13.5**	**13.5**	**13.5**	**13.5**	**13.5**	**11.3**
	(13.5)	(13.5)	(13.5)	(13.5)	(13.5)	(6.8–15.7)
NaCl (%; w/v)	**11.3**	**11.3**	**11.3**	**11.3**	**11.3**	**7.5**
	(11.3)	(11.3)	(11.3)	(11.3)	(11.3)	(3.8–11.2)
Urea (mol/L)	**0.8**	**0.8**	**1.0**	**0.8**	**0.8**	**0.8**
	(0.5–1.2)	(0.5–1.2)	(1.0)	(0.5–1.2)	(0.5–1.2)	(0.5–1.2)

As mentioned in the introduction, one of the criteria to investigate the efflux pumps investigated in this study was their reported connection to QAC resistance. It therefore comes as no surprise that the wild-type *E. coli* and *S. enterica* had significantly higher MIC values for each of the five QACs than the respective deletion mutants (Figure 1).

Although there were slight differences regarding the susceptibilities for the different strains and QACs, on average, the wild-type strains had a four times higher MIC compared to the deletion mutants with the highest observed for BC (6x) and the lowest for CP (2x). In conclusion, the results confirmed the influence of the studied efflux pumps on the susceptibility of the studied pathogens toward QACS. Consequently, the chosen bacterial strains can serve as models for studying the antimicrobial effect of ILs.

### Influence of Efflux Pumps on the Susceptibility to ILs

#### ILs With One Side Chain

**Figure 2:**
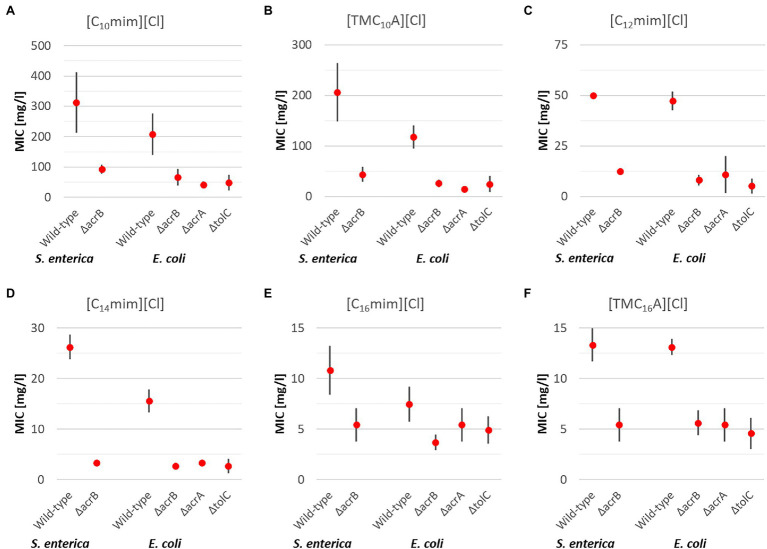
Mean MIC values for antimicrobial ionic liquids (ILs) with one elongated alkyl side chain [C_10_mim][Cl] **(A)**, [TMC_10_A][Cl] **(B)**, [C_12_mim][Cl] **(C)**, [C_14_mim][Cl] **(D)**, [C_16_mim][Cl] **(E)**, and [TMC_16_A][Cl] **(F)** including upper and lower limits of 95% CIs for the respective *S. enterica* and *E. coli* strains after 24 h incubation at 37°C.

Imidazolium ([C_n_mim][Cl]) and ammonium-based ([TMC_n_A][Cl]) ILs with varying alkyl side chain lengths were tested as representatives of ILs with one alkyl side chain (Figure 2).

In case of *S. enterica* wild type and the Δ*acrB* deletion mutant, for each of the six ILs with one elongated side chain, a significant higher resistance of the wild type compared to the deletion mutant was found. On average, the wild-type strain had a 4x higher MIC than the Δ*acrB* deletion mutant, with the highest observed for [C_14_mim][Cl] (8x) and the lowest for [C_16_mim][Cl] (2x).

In case, of *E. coli* very similar results for all three deletion mutants were obtained and will be discussed together. As was the case for *S. enterica*, the wild-type *E. coli* was significantly more resistant against ILs with one side chain than the efflux pump deletion mutants, all of which lack one gene necessary for a functional RND transporter. On average the wild type was able to withstand 4.3 times higher IL concentrations with the biggest differences observed for [C_12_mim][Cl] (6.5x) and the smallest for [C_16_mim][Cl] (1.7x).

Taken together for both bacterial species and all investigated efflux pump deletion mutants, the obtained results are quite similar to the results obtained for “classic” QACs.

#### ILs With Multiple Side Chains

**Figure 3:**
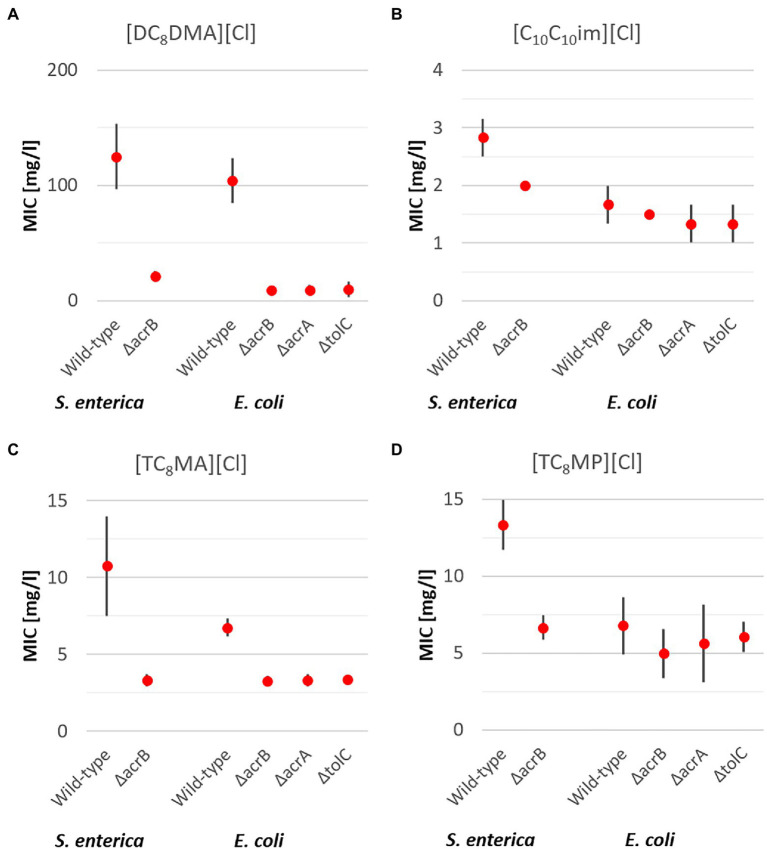
Mean MIC values for antimicrobial ILs with two elongated alkyl side chains [DC_8_DMA][Cl] **(A)** and [C_10_ C_10_im][Cl] **(B)** and three elongated alkyl side chains [TC_8_MA][Cl] **(C)** and [TC_8_MP][Cl] **(D)** including upper and lower limits of 95% CIs for the respective *S. enterica* and *E. coli* strains after 24 h incubation at 37°C.

In addition to antimicrobial ILs containing one elongated alkyl side chain, cations with multiple side chains have also been reported to have good antimicrobial activity and were thus included in the present study. The investigation included two ammonium-based ILs with two ([DC_8_DMA][Cl]) and three octyl side chains ([TC_8_MA][Cl]), one phosphonium based IL also with three octyl side chains ([TC_8_MP][Cl]), and one imidazolium based IL with two decyl side chains ([C_10_C_10_im][Cl]) and the general trends regarding IL antimicrobial activity were confirmed. With an increasing number of alkyl side chains of identical length, the antimicrobial activity is also increasing (Figures 2A, 3) which was observed for both bacterial specials and all strains. However, in case of the efflux pump deletion mutants, different results were obtained.

In case of *S. enterica*, the results for ILs with multiple side chains were found to be similar to those for ILs with one side chain and the QACs. For all ILs with multiple side chains, an average 3.1x times higher MIC of the wild type compared to the Δ*acrB* deletion mutant was found, with the highest observed for [DC_8_DMA][Cl] (5.9x) and the lowest for [C_10_C_10_im][Cl] (1.4x; Figure 3).

In case of *E. coli*, the results for ILs with multiple side chains were quite different for the different ILs and thus will discussed separately. In case of [DC_8_DMA][Cl], a significantly 11x higher MIC of the wild type was observed, demonstrating the impact of all three efflux pump types on the resistance against the IL. In case of [TC_8_MA][Cl], which has a similar cation core with one additional octyl side chain, a higher MIC (2x) for the wild type was observed in comparison with the three RND deletion mutants. Interestingly in case of [TC_8_MP][Cl], the MIC for all *E. coli* strains was around 6 mg/l with no significant differences between the four strains. Thus, demonstrating for the first time no significant effect on bacterial resistance against this antimicrobial active IL by the RND transporter. From these results, it seems as either the efflux pumps of *E. coli* are not able to remove the ILs from the cell inside, or the ILs have a different mode of action not acting inside the cell but for instance directly interact with the cell membrane as has been previously reported (Mester et al., 2016, 2019). Speaking against this hypothesis are the findings for *S. enterica*, as a clear and significant effect of the efflux pump was determined. As both bacterial species are Gram-negative bacteria with a similar cell membrane structure, it is more likely that the ILs act intracellularly and that the *E. coli* RND Transporter is less effective in transporting them out of the cell.

Similar results as for [TC_8_MP][Cl] were also found in case of the IL [C_10_C_10_im][Cl] containing two decyl side chains. In general, [C_10_C_10_im][Cl] was found to be the IL with the highest antimicrobial activity showing an average MIC of 1.8 mg/L, being even lower than the MICs of all QACs that are established biocides. Additionally, while all QACs as well as structurally similar ILs ([C_10_mim][Cl] and [DC_8_DMA][Cl]) are affected by the RND transporter in both bacterial species, for [C_10_C_10_im][Cl] no differences between the four *E. coli* strains and only a marginal effect for *S. enterica* (1.4x higher MIC) were found. These results demonstrate the possibility to design and obtain ILs that are not affected by multidrug efflux pumps belonging to the RND transporter type.

## Conclusion

This study aimed at investigating the effect of one of the most important multidrug efflux pump type belonging to the RND, which is associated with biocide and antibiotic resistance, in regard to the antimicrobial activity of ILs. By comparing the activity of antimicrobial ILs against both the wild-type strains and the respective efflux pump deletion mutants in two different bacterial species, the impact of the efflux pump could be directly assessed.

Investigating 10 different antimicrobial ILs with different cation structures enabled the identification of structure–activity relationships (SARs) in regard to efflux pump impact. The results of this study confirmed known SARs, such as the side-chain effect, for all tested strains as well as the general effect of efflux pumps. For all antimicrobial ILs with one elongated alkyl side chain, a clear and significant effect of the efflux pump in both species could be determined, regardless of the length of the side chain, the cationic head group, or the MIC. These results demonstrate that such ILs affect bacterial cells mostly intracellularly, where ILs have been reported to act in multiple ways including destabilizing proteins and hindering their enzymatic activity (Mester et al., 2019), increasing oxidative stress (Yu et al., 2016), or leading to DNA damage (Kowalczyk et al., 2018). In contrast, for ILs with multiple side chains, this study could identify for the first antimicrobial ILs that were not affected by the multidrug efflux pump. While for ILs with two and three octyl side chains ([DC_8_DMA][Cl] and [TC_8_MA][Cl]) the efflux pump significantly increased the MIC for both strains, in case of the structurally similar [TC_8_MP][Cl] no effect could be observed for *E. coli*. The antimicrobial IL that was found to be least affected by the efflux pump was [C_10_C_10_im][Cl], which was also the one with the highest antimicrobial activity of all investigated ILs, making it the most promising candidate as a novel biocide against multidrug-resistant bacterial species. At this point, it cannot be ultimately determined if the reduced effect of the efflux pump is either due to a different mode of action of these ILs, for example, a direct interaction with the cell membrane (Mester et al., 2016), or if the efflux pump is simply less effective to transport the ILs out of the cell. To further improve the antimicrobial activity, new ILs with the [C_10_C_10_im]^+^ cation as a lead structural motif can be designed and should be investigated against a broader set of resistant bacterial strains from clinical and environmental sources.

Overall, this study demonstrated the effect of efflux pumps belonging to the RND as an intrinsic defense mechanism of Gram-negative bacteria against antimicrobial ILs. The results demonstrate that by studying the effect of individual efflux pumps, structural motifs of antimicrobial ILs can be identified that are not affected by these efflux pumps. As this study focused solely on RND efflux pumps, future studies should include a more diverse set of efflux pump types to investigate possible similarities or differences in regard to the antimicrobial activity of biocidal ILs. Starting from the identified structural motifs in this study, the unique tuneability of ILs can be utilized for the development of effective biocides against MDR bacteria.

## Data Availability Statement

The original contributions presented in the study are included in the article/Supplementary Material; further inquiries can be directed to the corresponding author.

## Author Contributions

TG, PR, and PM: conceptualization. TG and PM: methodology. TG: formal analysis and investigation. PR and RK: resources. TG, PR, RK, and PM: data curation and writing—review and editing. TG and PM: writing. PM: supervision and project administration. PR: funding acquisition. All authors contributed to the article and approved the submitted version.

## Funding

The financial support by the Austrian Federal Ministry for Digital and Economic Affairs and the National Foundation of Research, Technology and Development is gratefully acknowledged.

## Conflict of Interest

RK is employed by Proionic GmbH.

The remaining authors declare that the research was conducted in the absence of any commercial or financial relationships that could be construed as a potential conflict of interest.

## Publisher’s Note

All claims expressed in this article are solely those of the authors and do not necessarily represent those of their affiliated organizations, or those of the publisher, the editors and the reviewers. Any product that may be evaluated in this article, or claim that may be made by its manufacturer, is not guaranteed or endorsed by the publisher.
