# Supplementary material for: Bacterial Resistance Toward Antimicrobial Ionic Liquids Mediated by Multidrug Efflux Pumps
Source: Front Microbiol. 2022 May 19;13:883931. doi: 10.3389/fmicb.2022.883931 (PMC9161554; doi:10.3389/fmicb.2022.883931)
Supplement: Supplementary file 1 [file Table_1.DOCX]

Supplementary Material

Bacterial resistance towards antimicrobial ionic liquids mediated by multidrug efflux pumps

Tobias Gundolf^1^, Roland Kalb^2,3^, Peter Rossmanith^3,4^, Patrick Mester^4*^

^1^ Christian Doppler Laboratory for Monitoring of Microbial Contaminants, Unit for Food microbiology, Department of Veterinary Public Health and Food Science, University of Veterinary Medicine, Vienna, Austria

^2^Proionic Production of Ionic Substances GmbH, Grambach, Austria

^3^Joint BioEnergy Institute, Lawrence Berkeley National Laboratory, Berkeley, CA 94720, USA

^4^ Unit for Food microbiology, Department of Veterinary Public Health and Food Science, University of Veterinary Medicine, Vienna, Austria

*** Correspondence:**Patrick Mester
Patrick-julian.mester@vetmeduni.ac.at

Table S1: Descriptions of all bacterial strains used in this study.

|  | Gene deletion | Alternative gene name | | Genotype, phenotype or selective markers | | Source | |  |
| --- | --- | --- | --- | --- | --- | --- | --- | --- |
| ***E. coli:*** |  |  | |  | |  | |  |
| BW25113 | wild-type |  | | △(araD-araB)567, △lacZ4787(::rrnB-3), λ- , rph-1, △(rhaD-rhaB)568, hsdR514 | | CGSC | |  |
| JW0451-2 | acrB | Multidrug efflux pump subunit AcrB | | △(*araD*-*araB*)567, △*lacZ*4787(::rrnB-3), λ^-^ , △*acrB*747::*kan, rph-1, △(rhaD-rhaB)568, hsdR514* | | CGSC | |  |
| JW0452-3 | acrA | Multidrug efflux pump subunit AcrA | | △(*araD*-*araB*)567, △*lacZ*4787(::rrnB-3), λ^-^ , △*acrA*748::*kan, rph-1, △(rhaD-rhaB)568, hsdR514* | | CGSC | |  |
| JW 5503-1 | tolC | Outer membrane protein TolC | | △(*araD*-*araB*)567, △*lacZ*4787(::rrnB-3), λ^-^ , △*tolC*732::*kan, rph-1, △(rhaD-rhaB)568, hsdR514* | | CGSC | |  |
| ***S. enterica:***  ATCC 14028s | wild-type | |  | |  | | (Horiyama et al., 2010; Yamasaki et al., 2013) | |
| NKS148 | acrB | Multidrug efflux pump subunit AcrB | |  | | (Horiyama et al., 2010; Yamasaki et al., 2013) | |  |

# REFERNCES

Horiyama, T., Yamaguchi, A., and Nishino, K. (2010). TolC dependency of multidrug efflux systems in Salmonella enterica serovar Typhimurium. *J. Antimicrob. Chemother.* 65, 1372–1376. doi:10.1093/jac/dkq160.

Yamasaki, S., Nagasawa, S., Fukushima, A., Hayashi-nishino, M., and Nishino, K. (2013). Cooperation of the multidrug efflux pump and lipopolysaccharides in the intrinsic antibiotic resistance of Salmonella enterica serovar Typhimurium. *J. Antimicrob. Chemother.* 68, 1066–1070. doi:10.1093/jac/dks528.
